# Generalizability of a Musculoskeletal Therapist Electronic Health Record for Modelling Outcomes to Work-Related Musculoskeletal Disorders

**DOI:** 10.1007/s10926-024-10196-w

**Published:** 2024-05-13

**Authors:** M. Wassell, A. Vitiello, K. Butler-Henderson, K. Verspoor, H. Pollard

**Affiliations:** 1 School of Computing Technologies, RMIT University, Melbourne, Australia; 2 School of Health, Medical and Applied Sciences, Central Queensland University, Queensland, Australia; 3 STEM|Health and Biomedical Sciences, RMIT University, Melbourne, Australia; 4 Faculty of Health Sciences, Durban University of Technology, Durban, South Africa

**Keywords:** Electronic health records, Occupational injuries, Occupational health physicians, Rehabilitation, Chiropractic, Musculoskeletal diseases

## Abstract

**Purpose:**

Electronic Health Records (EHRs) can contain vast amounts of clinical information that could be reused in modelling outcomes of work-related musculoskeletal disorders (WMSDs). Determining the generalizability of an EHR dataset is an important step in determining the appropriateness of its reuse. The study aims to describe the EHR dataset used by occupational musculoskeletal therapists and determine whether the EHR dataset is generalizable to the Australian workers’ population and injury characteristics seen in workers’ compensation claims.

**Methods:**

Variables were considered if they were associated with outcomes of WMSDs and variables data were available. Completeness and external validity assessment analysed frequency distributions, percentage of records and confidence intervals.

**Results:**

There were 48,434 patient care plans across 10 industries from 2014 to 2021. The EHR collects information related to clinical interventions, health and psychosocial factors, job demands, work accommodations as well as workplace culture, which have all been shown to be valuable variables in determining outcomes to WMSDs. Distributions of age, duration of employment, gender and region of birth were mostly similar to the Australian workforce. Upper limb WMSDs were higher in the EHR compared to workers’ compensation claims and diagnoses were similar.

**Conclusion:**

The study shows the EHR has strong potential to be used for further research into WMSDs as it has a similar population to the Australian workforce, manufacturing industry and workers’ compensation claims. It contains many variables that may be relevant in modelling outcomes to WMSDs that are not typically available in existing datasets.

## Introduction

Over the past twenty years, the cost of serious work-related musculoskeletal disorders (WMSDs) in Australia has increased by 58%, whilst time lost from the same disorders increased by 40% [1]. Globally, 26.44 million disability-adjusted life years are caused by work-related injuries [2].

Modifiable factors that have been shown to be associated with outcomes of WMSD include workplace cultural factors [3], social determinants [3, 4] psychosocial factors [4–6], physical and psychological demands of a job [3, 7], underlying health factors [8, 9], worker expectations [4, 5], self-efficacy [5], job dissatisfaction [4, 6] and psychosocial factors [6, 10]. Unfortunately, many of these modifiable factors are not collected in workers’ compensation claims databases, which are the key resources traditionally used for secondary research into outcomes for WMSDs [4, 11–14]. Often health plan and clinical intervention details cannot be obtained for analysis [15]. Longitudinal primary data collection can be expected to be required to address these gaps [16].

Electronic health records (EHRs) are digital versions of patient’s clinical notes, can collect longitudinal data on patients and are thus increasingly being used for research. EHR data have only recently started to be used in WMSD research [10, 17] and provide an opportunity to use real-world data for conducting research in cohort studies, randomized controlled trial studies or for building predictive models. Reuse of health care data is seen as valuable to address health care and research needs [18]. The information collected within EHRs includes many of the same demographic and injury details that are typically collected from claims databases, but they additionally provide much richer data on the details of patient care, modalities and interventions, underlying health and psychosocial factors, and other work variables. There is an opportunity to further research into WMSD outcomes through deidentified data collected from EHRs.

With demonstrated benefits of musculoskeletal (MSK) therapists working onsite in occupational health settings [19], there is the additional opportunity to collect data earlier, from the time of the first signs of pain or dysfunction, rather than at the time of an injury claim. This provides an opportunity for EHR studies to fill gaps in what is known about WMSDs from the time they occur to the time of claim [4, 10] or, indeed, to analyse what factors lead to claims, as patients are often only included in studies if they are completely off work for a period of time. Many WMSDs do not lead to a workers’ compensation claim or time off work yet may still have large costs associated with lost productivity and work ability. WMSDs that are not on workers’ compensation claims can become serious claims if factors affecting recovery are not addressed effectively. EHRs may provide early data on these pre-claim factors.

Due to the variety of MSK practitioners working in diverse settings and a lack of interoperable EHR systems, there are not yet consistent datasets from these practitioners that can be used for research into modelling outcomes of WMSDs. In addition, EHR data are often free text, which leads to challenges in extracting meaningful data due to the need for detailed chart review.

A specific criticism of many predictive models using EHR data is the relevance of the model external to the setting in which it was created [20]. Unfortunately, there is the potential for models to be bias towards demographics, particularly minority demographics, which can risk patient health outcomes [21]. Therefore, it is important to validate EHR data externally with respect to existing reference datasets [22] to determine whether the EHR dataset is relevant or to which populations it is generalizable to [23, 24] outside of the clinics where it was collected.

The data-generating organization (DGO) is a national onsite occupational health service operating in the private sector in Australia that has been collecting structured EHR data on MSK disorders for over 15 years. The DGO employs chiropractors, physiotherapists and osteopaths to treat workers in various industries. The DGO’s clients are workplaces that typically have higher job demands or repetitive work and the DGO operates in a value-based care model, where WMSD outcomes are closely monitored. The EHR collects data related to patient care, modalities used, and a vast amount of workplace psychosocial and cultural information that is relevant to workers recovery from injuries. The service collects information from the time of injury presentation and encourages early reporting of issues, which may be useful in providing a greater understanding of injury progression early on.

The EHR data could be valuable for future research or for modelling, such as for predicting outcomes of WMSDs if the dataset is relevant outside of its current setting, and broadly generalizable of national WMSD patterns. Hence, the aim of this study is to determine whether the EHR dataset contains a similar population of workers to Australian workforce and similar injury characteristics to workers’ compensation claims data. The EHR dataset has been reused for reporting of WMSD outcomes internally and externally for over 15 years: internally, such as providing analytics to clinicians to help them track outcomes, such as number of visits to resolve shoulder versus elbow injuries; externally, by reporting to workplaces about injury trends, such as by tracking mechanism of injury across departments or workplace cultural factors to cost of injury. The reporting led to the hypothesis that the EHR dataset contains workforce data similar to the Australian workforce and WMSD characteristics similar to the Australian workers' compensation claim characteristics for musculoskeletal (MSK) injuries. Describing the EHR dataset will provide evidence for its usefulness and generalizability for conducting further research into WMSDs and their outcomes.

Approval was obtained from the ethics committee of Central Queensland University, CQUHREC #0000023392.

## Methodology

### Study Design

Data should be assessed against the purpose for which it is to be reused, which in this instance is determining the relevance of the EHR dataset outside of its setting. Determining the relevance of the data is an important first step to ensure that any further research or models built from the data are generalizable. The EHR dataset has not yet been used for research, nor is it linked to existing registry datasets, such as workers’ compensation or occupational health registries. To determine the relevance of the EHR dataset for further research, Kahn’s harmonized terminology and framework for the secondary use of EHR data were utilized [22] where appropriate. The framework addresses the intrinsic data parameters including completeness and plausibility. It verifies these parameters with external sources or gold standards. Whilst the full analysis of the EHR dataset to this framework is outside the scope of this study, the relevant parts of the framework were applied. Completeness of each variable and any implausible values were validated. The EHR dataset was verified to determine its relevance in further study against the ABS and SWA datasets.

### Data Source

The EHR dataset is derived from an EHR used by musculoskeletal practitioners to treat workers with MSK disorders at occupational clinics nationally in Australia. The EHR is proprietary software built specifically to manage WMSDs. The data used for the analysis are from July 2014 to September 2021 after the EHR underwent significant upgrades in 2014. There are 57,570 musculoskeletal disorder records available for analysis and 20,663 unique patients seen across 10 industries and 101 sites. One patient may have suffered multiple WMSDs within the time frame. The data come from across seven of the eight states and territories across Australia in both rural and metropolitan settings. There are data from 59 chiropractors, eight physiotherapists and osteopaths. The EHR is highly structured, with minimal free text and many mandatory fields.

The musculoskeletal disorders (MSDs) contained in the dataset are those within the scope of practice for chiropractors, physiotherapists and osteopaths. Specifically, this excludes traumatic injuries requiring hospitalization and surgery, such as compound fractures. Non-musculoskeletal disorders such as respiratory and infectious diseases are also not managed at the clinics. The EHR dataset records visits to the health clinic within a workforce. The EHR dataset captures data from all MSK disorders and health conditions.

To protect patient privacy, the EHR dataset from the DGO was deidentified using globally unique identifier protocols, and then extracted by the organization from the relational database to a separate, secure location for the purpose of analysis as described in the TRANSFoRm Zone Model, which describes a process for dealing with data flow, privacy and confidentiality of personal patient data in research datasets [25]. Any potentially personal identifying information that was not required for the analysis was excluded from the dataset prior to extraction to further protect privacy, such as free text information. Access to the data required a secure login. Raw data were acquired in a.csv format.

To describe the EHR dataset and help determine the feasibility of the EHR dataset in further understanding workplace injuries treated onsite by musculoskeletal practitioners, two existing datasets were identified as criterion datasets, to help determine the relevance of the EHR dataset outside of current setting, the Australian Bureau of Statistics (ABS) Labour Force Survey data and the Safe Work Australia Workers’ Compensation claim data. Both of these criterion datasets contain aggregated and deidentified data.

The Australian Bureau of Statistics (ABS) Labour Force Survey data provide information about the labour market in Australia’s residents over 15 years of age and was used to determine the similarities between the Australian workforce and the workforce represented in the EHR dataset. It was chosen as it is the largest available dataset estimating Australian workforce characteristics. Variables that were assessed were industry, gender, duration of employment, age and nationality. These are key data inclusions that are important to identify population characteristics to allow for determination of similarities. The ABS and the EHR datasets have different purposes. The ABS workforce survey collects data about people in the workforce. The EHR dataset records data from people in the workforce that visit a health clinic for help with a MSK condition. ABS data were accessed through a publicly available database through the ABS website.

Safe Work Australia (SWA) collects data from all workers’ compensation claims lodged across Australia and is therefore the best dataset to use to determine similarities to the EHR dataset. SWA data were obtained from 1 July 2014 to 30 June 2020. The SWA dataset collects data on work-related MSK disorders; therefore, non-work-related MSK disorders were removed from the EHR dataset analysis. The SWA data were used to compare mechanism of injury, diagnosis and body region to the EHR dataset. SWA data were accessed from a request to SWA for publicly available data. Analysis against the SWA dataset is important as it is possible that the EHR dataset only contains minor injuries that may never be serious enough for a worker to take time off work or lodge a workers’ compensation claim, or that the EHR dataset only sees a small proportion of injuries within the specialty of the treating practitioners. It may also be that the setting of the onsite clinics influences the types of injuries seen and therefore not useful in hospital or medical practice settings.

### Variable Selection

Potential variables were identified through a literature review of variables associated with outcomes of WMSDs. The EHR subject matter experts were then consulted to determine further potential variables for analysis as outlined by Steyerberg [26]. From these, variables were selected based on the availability of overlapping variables in the EHR dataset and the ABS and SWA datasets. Whilst many other potential variables were present in the EHR dataset, the analysis was limited to those that could be compared to the external datasets.

The variables included are industry, age, gender, duration of employment and region of birth, which were assessed for similarities to the ABS data, and mechanism of injury, body location, diagnosis which were assessed for similarity to the SWA data. Geographical data were not available in the EHR dataset due to deidentification processes.

### Data Standardization

Dates for analysis of the EHR dataset were limited to the date ranges available from criterion datasets from 2014 to 2021.

The EHR dataset contains predominantly (99.3%) records of full-time workers, and therefore the ABS dataset was limited to full-time workers for analysis of workplace demographics. The SWA dataset obtained did not contain details of full-time employment status and therefore all employment status were included.

Industry was grouped by the Australian and New Zealand Standard Industrial Classification codes [27] in all three datasets, so no further standardization was required. Industry data were split into manufacturing and non-manufacturing. Age and duration of employment were grouped into categorical data using the grouping used by the ABS. Duration of employment recorded in the EHR dataset has some known data quality issues. Specifically, for a time, the EHR system rules set the date of employment to the date of the first appointment by default for new patients into the service. These records were identified and recorded as implausible values.

Data for nationality were aligned between EHR and ABS datasets. Further standardization was required according to EHR subject matter experts. Practitioners at the DGO report they often record a worker’s country of origin or cultural background within the nationality field, as this information was more clinically relevant than nationality. This difference leads to some inaccuracies in the nationality data. Secondly, within the EHR software, nationality defaulted to “Australia” prior to 2020 unless it was changed by the practitioner resulting in incorrect data so there was only a limited dataset for analysis. Due to this, nationality was grouped to the broader category that is used in the ABS dataset of region of birth.

The variable of mechanism of injury is recorded in the EHR as a mandatory field with several list options. These options did not completely align with the SWA dataset list options due to the SWA dataset recording non-MSK injuries and traumatic injuries that are not seen at the DGOs clinics. For this reason, it is not expected that mechanism of injury will be similar between the datasets. Non-MSK disorders were excluded from the SWA dataset. The WMSDs used for analysis included nature of injury/disease of “Traumatic joint/ligament and muscle/tendon injury” and “Musculoskeletal and connective tissue diseases”. The SWA dataset was then dichotomized into ‘body stressing’ or ‘non-body stressing’.

Body location data from SWA directly mapped to the EHR data. Both EHR and SWA datasets have a large list of potential diagnoses making standardization difficult. SWA uses an Australian coding system, which is based on the International Statistical Classification of Diseases and Related Health Problems (ICD) coding. The EHR diagnosis variable list is determined from conditions seen in the clinic and is not currently aligned with ICD or other coding systems. Six diagnoses were selected for analysis. These diagnoses were selected as they have tighter diagnostic criterion and are more likely to have pathoanatomical diagnosis and objective findings, rather than pain-based conditions that may not display pathoanatomical changes and are more subjective in diagnostic criteria, such as trigger points or lumbago.

### Data Analysis

Identification of the completeness of variables was determined using R statistical software v4.3.2 and reported as missingness. Potential reasons for missing values were summarized after consultation with EHR subject matter experts. The EHR subject matter experts were senior clinical leaders with experience in health informatics. Implausible values analysis was conducted to determine values that are outlying or likely to be incorrect based on local knowledge of the EHR subject matter experts, usually through assessing distribution analysis and by an understanding of potential system flaws and areas of potential clinician misuse of the EHR system.

EHR variables were analysed to determine percentage of records in each category and confidence intervals [23]. Confidence intervals were calculated to determine the limitations and stability of the results and therefore the confidence in the hypothesis.

The EHR dataset contains multiple WMSDs from a single worker over their career in the workforce. The ABS and SWA datasets also contain the same person multiple times, for example with two workers’ compensation claims. For this reason, it was more relevant to assess the EHR dataset at the WMSD event level rather than the person level.

The mean difference between the percentage of records for each variable analysis was recorded to provide a visual on the differences between the datasets. Mean difference confidence intervals were determined to be not appropriate for calculating as differences are expected as each dataset has a different primary purpose. For reporting, variables were reported as similar between the datasets if there was less than 10% difference between them. A result of over or under 10% however does not indicate that the EHR dataset should be reused. Models could be built with the EHR dataset with much wider variability; however, the population that the model is generalizable to may be diminished.

## Results

After standardization, most analysis involved 48,434 patient care plans across 10 industries. Records came from 101 workplaces from 2014 to 2021.

The database collects information relating to demographics, health history, injury details, examination, diagnosis, care plan, intervention and treatment details. Additionally, workers’ compensation details, advice given to employers, workers and patients, workplace modifications and work accommodations form part of the clinical record.

Physical job demands as well as workplace/job cultural, psychological and health demand factors are also collected through workplace assessments, and these are linked to the job that patients are conducting when injured. These fields are not used for this assessment but may be used in future analyses.

Obstacles to recovery or patient psychosocial “flags” [6] variables are recorded in structured format and have the potential to provide rich information in further studies. These include health factors, psychological factors, work beliefs, system and environmental factors [10, 28].

### Completeness Results

All variables besides industry were mandatory data capture, and therefore completeness was usually 100%, as shown in Table 1.

**Table 1:** Completeness of EHR dataset variables

Variable	Missing		Implausible		Total %
	*n* records	% records	*n* records	% records	
Industry	485	0.9	0	0	0.9
Age	0	0	0	0	0
Gender	0	0	0	0	0
Duration of employment	6073	10.6	10,071	17.5	28.04
Region of birth	0	0	0	0	0
Body location	0	0	0	0	0
Mechanism of injury	0	0	0	0	0
Diagnosis	0	0	0	0	0

A known quality issue existed with duration of employment, which meant that a default setting recorded injury date as the date of employment if not changed by practitioners, which is likely to be incorrect in all but the rarest cases. These were marked as missing and any subsequent care plans from these patients were recorded as implausible and all were excluded from the comparison to the ABS dataset when assessing of duration of employment only.

No other implausible values were identified, likely due to a well-structured EHR with rules around allowed values for each variable. Patients ages ranged from 14 to 77 years and duration of employment ranged from 0 days to 49 years.

Of the records included in the analysis, 95.4% were from the manufacturing industry.

Distributions of age, duration of employment, gender and region of birth were similar between the EHR dataset and ABS dataset as seen in Table 2. There were more females in the manufacturing industry in the EHR dataset than in the Australian workforce population (9.5%). There were also less employees with over 10 year’s service in the EHR (9.1–12.4%).

**Table 2:** Comparison of EHR dataset to ABS labour force survey datasets

Variable	Industry and breakdown	EHR dataset			ABS dataset				Mean difference (%)
		*n* records	% Records	95% CI	*n* by ’000 records		% Records	95% CI	
Age	*All industries*	48,434			58,496				
	15–24	5142	10.60	10.58–10.62	5481		9.38	9–9.82	1.22
	25–34	12,381	25.46	25.43–25.49	15,469		26.45	25.9–27.02	−0.99
	35–44	12,752	26.35	26.34–26.36	13,915		23.78	23.31–24.27	2.57
	45–54	11,245	23.35	23.32–23.39	13,299		22.74	22.31–23.12	0.61
	55–64	6291	12.97	12.94–13	8678		14.83	14.44–15.22	−1.86
	65 +	621	1.27	1.24–1.29	1647		2.81	2.59–3.03	−1.54
	*Manufacturing*	46,227			5231				
	15–24	5704	12.68	12.50–12.85	487		9.35	7.42–11.28	3.33
	25–34	12,048	26.42	26.3–26.53	1138		21.82	18.69–24.95	4.6
	35–44	11,829	25.52	25.49–25.55	1269		24.33	21.6–27.1	1.19
	45–54	10,430	22.22	22.11–22.33	1321		25.31	22.65–28.07	−3.09
	55–64	5675	12.01	1.11	863		16.53	14.4–18.66	−4.52
	65 +	541	1.15	1.2	140		2.66	1.7–3.62	−1.51
Gender	*All industries*	48,434			58,489				
	Female	15,731	32.54	32.52–32.56	21,323		36.44	35.61–37.33	−3.9
	Male	32,703	67.46	67.17–67.74	37,166		63.56	63.54–63.56	3.9
	*Manufacturing*	46,227			5212				
	Female	13,929	30.13	28.8–31.45	1074		20.59	18.12–23.3	9.54
	Male	32,298	69.87	67.86–71.89	4138		79.41	73.96–84.88	−9.54
Region of birth	*All industries*	15,469			17,146				
	Americas	354	2.29	2.28–2.3	309		1.73	1.46–2.02	0.56
	Australia	10,700	69.23	29.17–29.29	11,432		67.00	65.53–68.48	2.23
	N. Africa and Middle East	339	2.19	2.03–2.36	268		1.48	1.22–1.76	0.71
	North-East Asia	992	6.40	6.35–6.45	652		3.71	3.31–4.13	2.69
	North-West Europe	116	0.75	0.72–0.78	1173		6.77	6.32–7.22	−6.02
	Oceania and Antarctica	853	5.50	5.43–5.56	666		3.95	3.58–4.34	1.55
	South-East Asia	1297	8.36	8.28–8.43	815		4.79	4.35–5.22	3.57
	South and Central Asia	425	2.74	2.71–2.77	1094		6.20	5.6–6.81	−3.57
	South and East Europe	76	0.49	0.48–0.51	356		2.07	1.78–2.36	−1.58
	Sub-Saharan Africa	317	2.05	2.05–2.05	372		2.26	1.96–2.55	−0.21
	Inadequately described	0	0.00	0.0	9		0.00	^a^	0.0
	*Manufacturing*	14,033			1502				
	Americas	348	2.50	2.5–2.5	24		1.65	0.85–2.44	0.85
	Australia	9427	67.17	67.1–67.2	946		63.64	58.11–69.13	3.53
	N. Africa and Middle East	335	2.39	2.2–2.6	25		1.53	0.82–2.33	0.86
	North-East Asia	947	6.75	6.7–6.8	51		3.02	2.02–4.2	3.73
	North-West Europe	102	0.73	0.7–0.8	102		6.63	5.05–8.33	−5.9
	Oceania and Antarctica	821	5.85	5.8–5.9	66		4.52	3.22–5.83	1.33
	South-East Asia	1260	8.98	8.9–9.1	115		7.92	6.15–9.81	1.06
	South and Central Asia	410	2.92	2.9–3.0	102		6.69	4.93–8.47	−3.77
	South and East Europe	71	0.51	0.5–0.5	43		2.71	1.69–3.77	−2.2
	Sub-Saharan Africa	312	2.22	2.2–2.2	29		1.70	0.98–2.87	0.52
	Inadequately described	0	0.00	0.0	0		0.00	0	0.0
Duration of Employment	*All industries*	32,290			58,503				
	Less than 6 months	3997	12.48	12.43–12.54	5033		8.59	8.26–9.21	3.89
	6–11 months	2859	9.07	8.99–9.16	4991		8.52	8.03–9.05	0.55
	1 year	4871	15.43	15.31–15.54	5194		8.88	8.43–9.46	6.55
	2 years	4236	13.34	13.25–13.43	6056		10.34	9.81–10.95	3.0
	3–4 years	5916	18.42	18.29–18.54	8588		14.67	14.04–15.34	3.75
	5–9 years	7177	21.83	21.74–21.92	11,733		20.08	19.32–20.86	1.75
	10–19 years	2569	7.47	7.30–7.65	10,421		17.80	17.1–18.59	−10.33
	20 years and over	665	1.96	1.83–2.08	6487		11.09	10.6–11.59	−9.13
	*Manufacturing*	30,820			5230				
	Less than 6 months	3839	12.52	12.46–12.57	434		8.26	6.78–10.37	4.26
	6–11 months	2588	8.56	8.49–8.63	395		7.56	6.05–9.15	1.0
	1 year	4344	14.66	14.55–14.78	433		8.26	6.75–10.32	6.4
	2 years	3853	12.55	12.46–12.63	487		9.34	7.52–11.31	3.21
	3–4 years	5632	18.61	18.48–18.73	735		14.07	11.77–16.45	4.54
	5–9 years	7320	23.38	23.31–23.46	1042		19.93	17.16–22.85	3.45
	10–19 years	2631	7.88	7.69–8.06	1061		20.29	17.91–22.78	−12.41
	20 years and over	613	1.84	1.71–1.97	643		12.29	10.37–14.37	−10.45

^a^ABS data not available due to limited records

In analysing similarities from the EHR dataset to the SWA dataset, upper limb WMSDs were more prevalent (12.3–16.4%) and lower limb WMSDs were less prevalent (13–17.3%). Diagnoses were similar between the EHR and SWA data but limited to low percentages of records due to the selection of diagnoses analysed (14.3–15.7%). A mechanism of injury of body stressing was much more prevalent in the EHR dataset (26.2–32.9%) due to the nature of the injuries seen in the clinic (Table 3).

**Table 3:** Comparison of EHR dataset to SWA workers’ compensation claims dataset

Variable	Industry and breakdown	EHR dataset			SWA dataset^a^			Mean difference (%)
		*n* records	% Records	95% CI	*n* records	% Records	95% CI	
Mechanism of injury	*All industries*	31,996			870,851			
	Body stressing	29,411	91.85	91.77–91.93	516,750	58.93	58.93–58.93	32.92
	Repetitive overuse	15,865	49.00	48.84–49.15				
	Static postural	2849	8.97	8.94–9				
	Nothing specific	2684	8.45	8.42–8.48				
	Exertional overuse	8013	25.44	25.37–25.5				
	Traumatic	2585	8.15	8.13–8.18				
	*Manufacturing*	29,082			88,452			
	Body stressing	26,849	92.26	92.2–92.33	58,419	66.02	66.02–66.03	26.24
	Repetitive overuse	15,055	51.04	50.9–51.19				
	Static postural	2287	7.94	7.927.96				
	Nothing specific	2210	7.68	7.72–7.76				
	Exertional overuse	7297	25.60	25.53–25.66				
	Traumatic	2233	7.74	7.72–7.76				
Bodily location	*All industries*	31,996			870,779			
	Head and neck	4925	15.37	15.34–15.4	35,709	4.07	4.07–4.07	11.30
	Upper limbs	15,705	49.03	49.01–49.05	28,258	32.67	32.67–32.67	16.36
	Trunk	8686	27.22	27.21–27.24	282,467	32.09	32.08–32.09	−4.86
	Lower limbs	2418	8.38	8.37–8.39	221,919	25.71	25.71–25.71	−17.33
	Multiple and unspecified	0	0.00		48,026	5.45	5.45–5.46	−5.45
	Manufacturing	29,082			88,442			
	Head and neck	4163	14.21	14.19–14.23	3462	3.25	3.25–3.25	10.96
	Upper limbs	14,794	50.87	50.85–50.89	40,788	39.05	39.04–39.06	11.82
	Trunk	7827	27.01	27–27.03	35,292	33.01	33.02–33.03	−6
	Lower limbs	2298	7.91	7.9–7.91	22,106	20.99	20.99–21	−13.08
	Multiple and unspecified	0	0.00		4030	3.68	3.66–3.71	−3.68
Diagnosis	All industries	31,996			870,779			
	Tendonitis/opathy/osis	1790	5.59	5.38–5.81	11,692	2.63	2.63–2.63	2.97
	Disc disease	613	1.92	1.76–2.07	13,076	2.33	2.33–2.33	−0.41
	Bursitis	428	1.34	1.14–1.54	6893	1.60	2.59–2.6	−0.26
	Epicondylitis/opathy	805	2.52	2.32–2.71	4310	1.21	1.21–1.22	1.30
	Synovitis and tenosynovitis	449	1.40	1.22–1.59	3535	0.82	0.82–0.82	0.58
	Ganglion, trigger finger and Dupuytrens	506	1.58	1.41–1.75	893	0.21	0.21–0.21	1.38
	*Manufacturing*	29,082			88,451			
	Tendonitis/opathy/osis	1786	5.67	5.54–5.79	1858	3.59	3.59–3.59	2.08
	Disc disease	578	1.83	1.81–1.85	1615	2.47	2.46–2.49	−0.64
	Bursitis	406	1.31	1.28–1.35	886	2.02	2.01–2.03	−0.71
	Epicondylitis/opathy	809	2.59	2.53–2.65	768	1.83	1.82–1.85	0.76
	Synovitis and tenosynovitis	467	1.51	1.45–1.57	481	1.00	1–1.01	0.51
	Ganglion, trigger finger and Dupuytrens	525	1.71	1.56–1.85	235	0.46	0.46–0.46	1.25

^a^SWA total records vary between categories due to suppressed data for low records in SWA dataset

## Discussion

### The Analysis

Completeness is a common problem in EHR studies and whenever data are used for secondary analysis. The EHR dataset demonstrates high completeness compared to many similar studies [29–32]. Completeness in the EHR in this study is a consequence of mandatory data capture in many fields. This, however, leads to other types of errors and potential bias [33]. Practitioners may, for instance, always select the same list options for every patient. As EHRs are becoming more structured in data collection, assessment of practitioner’s individual data entry will become crucial, rather than simply relying on completeness findings. Further analysis of individual practitioner data entry in this EHR dataset has been previously described [34]. The completeness findings also demonstrate the importance of subject matter expert knowledge to understand where potential data quality issues exist.

Whilst the confidence intervals are acceptable, there is known variability in WMSDs in different industries [35]. Even within a single industry, there can be high variability due to the specifics of each workplace. For example, local populations vary as regional locations have different migrant populations. Even within an industry, the job demands can vary due to factors such as automation. Workplace hiring policies impact the specifics of the population at each workplace. Perhaps most importantly, workplace cultural factors and injury management program details all impact who attends onsite health clinics. On top of the factors external to the DGO, internal organizational factors such as practitioner training and clear definitions around data input requirements impact confidence intervals. Additionally, confidence intervals will be affected by the clinical and operational governance processes in the DGO as well as many other data quality parameters outlined in frameworks specific for the reuse of EHR data [22, 36]. Within the DGO in this study, there is already reuse of the EHR data for reporting outcomes to workplaces and clinicians. For example, practitioners in the organization have metrics and governance processes for reviewing clinical notes for clinical quality reasons. Failure to report on organization practices can lead to issues with external and internal validity of studies and models [37].

### The Variables

The EHR dataset was found to contain records predominantly from the manufacturing industry. Manufacturing is usually found to have an increased risk of poor outcomes and long-term disability compared to many industries [16, 38, 39]. So, whilst the dataset was not shown to be representative of industries across the Australian population, there is a need for investigation into higher rates of disability and poor outcomes for the manufacturing industry.

The EHR dataset demonstrated a similar distribution to ABS data for age groups and duration of employment. The EHR dataset has more representation in younger age groups. This is likely to be due to the types of workplaces that the EHR dataset represents. The DGO typically works at employers that have high risk and heavy manual roles. These employers often employ international working holiday visa holders who are younger in age and have shorter employment lengths. These international workers are excluded in the ABS dataset. The findings describe the physical outcomes workers may experience in workplaces with high manual labour roles. In the authors’ experience, older age and long-term workers tend to self-select for alternate employment due to the impact of years of hard labour on their bodies, which is supported by the literature finding that older injured workers are less likely to return to work and suffer long-term disability [38–40]. These variations may have implications on further study of the EHR dataset and affect the generalizability which may need to be accounted for in models built from the data.

Gender is not completely comparable between datasets and differences are likely to be partially due to the population studied, assessment of working hours or how they are broken down for analysis. Gender analysis would be improved with specific recording of sex and gender allowing for diversity.

The EHR dataset demonstrates a slightly higher rate of Australian workers (2.2–3.5% mean difference) than the ABS data. This could be expected as ABS data exclude international residents as previously discussed. Many regions of birth are represented in the EHR dataset which may be useful in further research to analyse cultural and genetic physical differences in types and response to WMSDs. For example, the average height of a Burmese male is 164.7 cm versus an Australian male is 175.6 cm. Outcomes to injury analysis may need to consider work modifications such as bench heights as factors influencing recovery.

The EHR dataset was unable to be completely compared to the SWA dataset due to differences in reporting categories. Comparing the ‘body stressing’ category found that the EHR dataset reporting around 92% of complaints as ‘body stressing’ compared to SWA data of 58.9–66%. The SWA dataset would include many conditions outside of the scope of practice of the onsite health service, such as those requiring surgical intervention, likely explaining the variability. Non-traumatic injuries, such as those involving repetitive mechanisms, are often found to be more likely to lead to poor recovery [4, 16, 39, 40]. Repetitive movement mechanisms represent 37% of the injuries within the EHR dataset providing an opportunity for further analysis into factors leading to poor outcomes within this population.

The EHR dataset contained more upper limb MSDs than the SWA dataset, with less lower limb and trunk MSDs. Body region coding is often subjective. Clinician may record area of pain or the area related to the underlying cause of the problem. Neck and back injuries are commonly reported as having worse outcomes for long-term disability [38, 41] and are well represented in the EHR dataset.

There were six diagnosis categories analysed, with similar rates of diagnosis within the EHR dataset and SWA dataset, although percentage of records were low in each diagnosis. Variation is expected as there are many challenges with diagnosis. Practitioners tend to diagnose MSK disorders either by the pathoanatomical lesion or by the potential causative nature. Interpractitioner reliability of tests used to diagnose is often questionable [42, 43] and skill level of practitioners likely plays a role.

### The EHR Opportunities

Lack of employment secondary to health issues has negative consequences on health [44, 45] just as early returning to work has benefits [46]. A strong predictor of long-term disability is days until medical care is received for a work-related injury [38]. Staying at work with coordinated care [4] and appropriate work modifications is better for return to work, reduced costs and more positive outcomes [47, 48]. EHRs used by onsite clinics can collect data from the time an injury occurs and create the opportunity to develop early predictors to determine who is more likely to stay at work or intervene early to reduce the risk of poor return to work with coordinated care. This has been demonstrated by early collection of the single-item Work Ability Index which can predict the risk of long-term disability [44]. Recording of known psychosocial, social determinants, job demands, work beliefs and environmental/system factors through EHRs needs to occur as early as first signs of pain, dysfunction or loss of work ability to be able to offer early intervention, even prior to claim submission.

This study helps to demonstrate the value of the EHR dataset for reuse in the wider population. Whilst the comparison was to Australian datasets, the study is relevant globally as many countries such as Canada, United States, United Kingdom and India operate similar workers compensation systems. There is an opportunity with the adoption of EHRs to develop EHR WMSD registries to allow for better research into WMSDs.

Checking the relevance and generalizability of the data is an important step to understand potential further uses for the dataset. When implementing predictive machine learning models, the predictive value of a machine learning model reduces as the model is applied in different settings [49] and checking the relevance of the data may provide a better understanding of whether the model should be used in different settings. Population bias is a known issue with many EHR machine learning models [21]; for example, models often fail to accurately represent all nationalities due to lack of diversity in training sets. This research paper helps provide an understanding of the generalizability of the EHR dataset so that any lack of diversity or data deficiencies can be understood [50] prior to further research and guide development of appropriate research questions.

The study demonstrates that this DGO real-world dataset derived from musculoskeletal practitioners can be used to advance WMSD outcomes, advocating for chiropractors, physiotherapists and osteopaths in treating and managing WMSDs with and without workers’ compensation claims. The research is important in setting a benchmark of what is achievable when using EHRs, as currently, EHR data from allied health practitioners are challenging to collate, due to practitioners working in multiple disparate setting on different systems.

## Strengths and Limitations

The strength of the study is the ability to provide an important methodological step in assessing the suitability of an EHR built to manage WMSD for research which has the potential to improve predictive modelling such as machine learning models built on EHR data. Demonstrating the generalizability of the data reduces risks of bias in models and increases the chances models can be used broadly. The EHR collects structured data on psychosocial factors and workplace factors such as workplace culture, which are often not available in workers’ compensation claims databases or registry data.

One EHR is not representative of all EHRs or all practitioners or professions. The study is limited to a single EHR dataset of workers who presented for care to one organization and is not necessarily representative of the entire workforce at a worksite.

Whilst this study demonstrates that the EHR dataset contains similar data to ABS and SWA datasets, this does not mean that a model produced from the dataset would be valid across all MSK occupational health care organizations as further data quality analysis is required first. Modelling would also need to assess the methods used in this study to determine if by assessing the EHR dataset at the person level leads to overrepresentation of specific types of workers, such as those that are likely to suffer more WMSDs.

Whilst the study does not report on many of the variables that are collected within the EHR, it accurately determines an important first step, that the EHR dataset is similar to workforce characteristics and workers’ compensation claims statistics in the industries it works with.

A specific framework for the assessment of data quality with EHRs for secondary research [22] has been used as the terminology and framework for this paper in effectively determining missingness and external verification of the EHR dataset; however, the paper does not attempt to determine the overall data quality of the EHR. Further studies should conduct a complete data quality assessment in line with the recognized frameworks to determine if the dataset is appropriate for use in future research or building predictive models into outcomes of WMSDs.

## Future Research

The study was unable to analyse many variables that have been shown to be important in understanding outcomes to WMSDs such as obstacles, work modifications, job demands, psychosocial and health interventions as no comparable data are available in the national datasets. However, these variables are available for analysis within the dataset and will be analysed in further data quality analysis studies to determine the reliability of the data for predicting outcomes to WMSDs. The opportunity for further study on many important factors that are contained within this dataset offers potential for improving WMSD outcomes.

## Conclusion

The study describes an extensive real-world data collection from chiropractors and occupational musculoskeletal professionals that can potentially be a valuable dataset for further research into WMSDs. The EHR collects many variables known to be predictors in determining outcomes of WMSDs and that are traditionally difficult to collect, such as clinical care details, health and psychosocial factors, job demands and workplace cultural factors. The analysis of the EHR dataset demonstrates that it is similar in many ways to comparative datasets from SWA and ABS. It can be considered to be broadly representative of the Australian workforce, manufacturing industries and Australian workers' compensation claims. The EHR dataset represents a wide range of patient musculoskeletal disorders from many age groups, regions of birth, body regions and diagnoses. The EHR dataset demonstrates high completeness due to structured and mandatory data capture. Overall, the analysis suggests that the EHR will support meaningful research and can contribute to reducing the costs and impact of WMSDs.
